# Dialogue as a tool of nutrition literacy in an agricultural intervention programme in Odisha, India

**DOI:** 10.1186/s43170-022-00090-x

**Published:** 2022-05-10

**Authors:** Rama Narayanan, Akshaya Kumar Panda, D. J. Nithya, R. V. Bhavani

**Affiliations:** 1 Independent Consultant, Community Nutrition, Chennai, India; 2 M S Swaminathan Research Foundation, 3rd Cross Street, Institutional Area, Taramani, Chennai, 600113, India; 3 Consultant, FAO, Chennai, India

**Keywords:** Nutrition literacy, Dialogue, Nutrition sensitive agriculture, Behaviour change

## Abstract

**Background:**

An ongoing action research nutrition literacy programme based on Freire’s approach of raising critical consciousness through the use of dialogue as a pedagogic tool is being implemented as part of a nutrition sensitive agricultural intervention in tribal Odisha. One hundred and eight adults, referred to as Community Hunger Fighters (CHFs) underwent two modules of a residential training programme of two and a half days each, spread over two months. Through discussion they explored the reasons behind the lack of diversity in their daily diets and identified the social, economic and cultural barriers to food intake in the context of their own poverty. They undertook collective exercises in nutrition sensitive agricultural planning. The transformative behaviour of the CHFs was captured through observation, interviews and focus group discussion with a set of qualitative indicators.

**Results:**

The methodology of dialogue as a pedagogic tool generated a discussion about food security among the community. CHFs identified key messages and shared them with fellow villagers in imaginative ways. The process of critical reflection and analysis helped understand gender disparities, the bottlenecks in food production, brought in life style changes to improve food intake and created a demand for technical training for improving agricultural productivity. Thirty eight had started a nutri-garden and several took on leadership roles on other issues of importance besides food security.

**Conclusion:**

Dialogue as a pedagogic tool for nutrition literacy in an agricultural intervention programme has the potential to facilitate a process of critical reflection on the socio cultural and economic barriers to food production and consumption thereby leading to transformative action.

**Supplementary Information:**

The online version contains supplementary material available at 10.1186/s43170-022-00090-x.

## Background

Nutrition communication has been an integral part of health and nutrition interventions. Though nutrition services have improved the nutritional status of the targeted population, they have either not had the desired effect on dietary habits (Bull et al. 2014) or even if they did, their long term sustainability is not yet established (Black et al. 2017). According to Nutbeam (2000) health programmes that relied primarily on transmission of information without paying attention to the social and economic conditions of the participants failed to bring about sustainable changes in health behaviour. Distinguishing between ‘education’ and ‘literacy’, he considers literacy as a composite term to describe a range of outcomes to health education and communication activities. He identifies three levels of health literacy. Functional literacy that involves transmission of basic health knowledge, with limited goals conforming to prescribed actions. This approach lacks interactive communication and does not support individual autonomy. The next is interactive literacy that enables individuals to derive a meaning from health information and apply it in their daily lives. It supports development of personal skills, self confidence and motivation, leading to behaviour change. The third level is critical literacy that focuses on empowering individuals to question and critique information and practices so as to gain more control over life, events and situations. The latter has subsequently been applied in varied health and nutrition settings.

The integration of critical literacy in school health programmes emerged in Europe and North America in the mid 1980’s. It challenged the traditional top down approach of imparting information. It looked beyond the curriculum and paid attention to other domains such as school environment, school health policies, links with health services and partnership with the local community. This approach shifted health education into a new dynamic and political domain. It strove to improve students’ skills in advocacy and help them achieve a sense of empowerment. The new framework facilitated health literacy at all three levels proposed by Nutbeam and was in place in most European countries by early 1900 (St Leger 2001).

Brown (2015) describes the experience of introducing adult learning principles in a nutrition education programme for Mexican American women offered through adult education centres. The extension educators were challenged with providing a culturally sensitive nutrition education programme for weight loss to the participants. The design included a three-prong approach of programme planning, teaching and learning and research. A pilot study revealed that women wanted more time in the classroom for socialization and developing relationships. They did not like cooking unfamiliar foods and felt the classes offered more information than needed. A second series of classes were planned for 7 weeks at the end of which 15 women who attended 50% of the classes lost 35 cumulative pounds. They were also willing to share their learning with their counterparts in the community.

Students of a community college were involved in an interdisciplinary study of food and agriculture while simultaneously being involved in actual food production for low income members of their community (Adelman and Sandiford 2007). By growing and distributing food, students learnt about the extent of poverty and hunger prevailing in their own communities. They understood that the widespread use of synthetic chemicals by way of fertilizers and pesticides found in the foods that they ate and released into the environment was a threat to humanity. They were convinced that organic agriculture was possible and food security could be addressed at the local grassroots level. They got involved in the national alternate agriculture movement, rooted in local decision making, civic action and freedom from the corporate food industry.

The empowerment model of literacy requires a multidisciplinary approach drawing from the domains of education, development, communication and social sciences. This also raises the question of what pedagogic strategies may be adopted to raise critical thinking especially in resource poor settings with low formal literacy. The Brazilian educator Paulo Freire (1994) used dialogue as a pedagogic tool to raise the ‘critical consciousness’ of people with no formal literacy skills, that led to empowered achievements and outcome. Subsequently others who followed in his footsteps (Wallerstein and Bernstein 1988; Naiditch 2010) have also attributed to sustainable outcomes. A nutritional literacy programme in India modelled on Freire’s principles has reported that besides individual transformation towards healthy eating, participants could expand the scope of nutrition security from beyond food to one of healthy lifestyle such as giving up of tobacco and alcoholism (Narayanan and Rao 2019). Through group exercises, they reflected on the fact that during peak agricultural season they usually skipped meals. They sold the good quality grains and retained the lower quality ones for consumption. This helped them realize that they did not pay much attention to their health and were only filling their stomachs. From gaining access and control over forests to bringing in a variety of foods for cultivation, participants identified ways of improving food security. According to Gavaravarapu (2019) in India, only a small proportion of community nutrition research is devoted to nutrition education. Though there are a few scattered efforts in experimenting with newer communication approaches, there is a dearth of published literature. The paper discusses an action research programme on nutrition literacy using dialogue as a pedagogic tool to raise critical consciousness. It is being implemented by the NGO, MS Swaminathan Research Foundation and funded by the Department of Agriculture and Farmers’ empowerment, Government of Odisha under *Rashtriya Krishi Vikas Yojana* (RKVY) as part of an agricultural intervention programme in Eastern India. Since the project is ongoing, the paper presents the conceptual basis and methodology of implementation with some early observations from the field.

### Conceptual basis

#### Definition of nutrition literacy

Krause et al. (2018) observe that, definitions of nutrition literacy were limited to describing the abilities needed to obtain and understand nutrition information. The International Union of Nutrition Sciences (IUNS) and the World Health Policy Forum in a joint initiative stress the need for an interdisciplinary approach and define Nutrition science ‘as the study of food systems, foods and drinks, and their nutrients and other constituents; and of their interactions within and between all relevant biological, social and environmental systems’ (Cannon and Leitzmann 2006). Hence an operational definition of nutrition literacy proposed in this project is ‘the process by which individuals/communities are empowered to critically analyse their nutrition situation and engage with existing social, cultural, biological, political and environmental realities meaningfully to achieve dietary diversity and have access to safe drinking water, sanitation and adequate health services in order to optimize nutrition outcomes’.

#### Embedding nutrition literacy in agricultural intervention

Nutrition sensitive agriculture is a food based approach to agriculture development that puts nutritionally rich foods, dietary diversity and food fortification at the heart of overcoming malnutrition and micronutrient deficiencies (FAO 2014). This involves addressing existing farming systems to be nutritionally sensitive. Farming System for Nutrition (FSN) is defined as ‘the introduction of agricultural remedies to the nutritional maladies prevailing in an area through mainstreaming nutritional criteria in the selection of the components of a farming system involving crops, farm animals and wherever feasible fish’ (Nagarajan et al. 2014). Embedding a nutritional literacy component in an agricultural intervention involves focusing on the opportunities available and challenges involved in the production and consumption of nutritious foods. Hence with due acknowledgement to the non-food factors involved in determining nutritional outcomes, the nutrition literacy project delimited itself to addressing crop and food related issues within the social, economic, political and environmental context of the participants.

#### Communication strategies

Behaviour change communication (BCC) is a research-based consultative process of addressing knowledge, attitudes and practices of participants using a mix of interpersonal, group and mass-media channels, including participatory methods (UNICEF ROSA 2005). According to Nancy and Dongre (2021) BCC evolved gradually from health education and Information, Education and Communication (IEC). Several theories and models operating at the individual, interpersonal and community levels have been put forward to explain the core constructs of BCC. The approach of BCC is to develop communication strategies to promote appropriate positive behaviour and facilitate a supportive environment for people to initiate and sustain such behaviour. This concept is further expanded to social marketing (i.e.) ‘the application of marketing principles to enable individual and collective ideas and actions in the pursuit of effective, efficient, equitable, fair and sustained social transformation’ (Saunders et al. 2015) wherein the marketer becomes a facilitator and participant of change rather than being a behavior change agent.

The focus of BCC is individual behaviour change and does not take into account the social determinants. Individual behaviour change must be accompanied by social transformation such that power is distributed within various social and political institutions, paving the way for Social Behaviour Change Communication (SBCC) (UNICEF 2005). The ‘S’ in SBCC indicates that individuals and their social relationships are determined by larger structural and environment systems, such as gender norms, power hierarchies that include class and caste, cultural practices, the social, organizational and political atmosphere and local economy (Kumar 2020). SBCC adopts a socio ecological model integrating all the above aspects considered essential to address barriers and opportunities for social and behaviour change and to promote sustainable solutions (McKee et al. 2014). It is a process that is built on three key strategies—communication, behaviour change and social change.

The crux of SBCC’s implementation is dialogue, discussion and negotiations. Paulo Freire, the Brazilian educator, was a pioneer in the use of dialogue in adult literacy. Freire’s pedagogy recognizes that adult learners possess unique knowledge and it should be co-constructed and investigated with all participants; to enable this the classrooms should be democratic and learners must be given an opportunity to speak and break the ‘culture of silence’. They should be engaged in a critical dialogue to raise awareness about their own situation and given hope that they can change it. This process is called ‘conscientization’ where individuals recognize their potential and take action according to their new understanding. Dialogue is a co-operative activity involving respect and is linked with informed action and critical reflection (Freire 2005). The action reflection process enables learners to perceive the social, economic and political contradictions and to act in the context of these realities.

Bonatti et al. (2021) investigated how social learning and Freire’s key concepts were manifest in two case studies of food security in Brazil. The two initiatives were a community seed bank in the northeast of the country and distribution of biodiversity kits in the south. A mixed method approach was used based on semi-structured interviews and literature review. The community seed bank initiative at first maintained stocks of corn and beans that contributed to the conservation of local species and cultivars. A family could borrow a certain quantity of seed and repay it later after harvest plus a small percentage as decided by the community. The seeds network was a partnership between a local NGO and farmer organizations that had accumulated local knowledge. Through meetings farmers identified the seeds to be multiplied and places to do so. Agro ecological management was based on the knowledge of local farmers which included storage and silo making. The farm families promoted knowledge exchange through fairs, experimentation and the ‘Seeds of Passion’ cultural festival. Through these activities they shared the values and cultures of their land and sought dialogue with public policies and legal backing for family agriculture. The second initiative addressed the issue of disappearing landraces. A biodiversity kit consisting of local landraces was created through a participatory process involving local farmers, technicians and scientists. In the implementation phase, small holder farmers met with technicians in formal and informal meetings where they explained their food production process and articulated their demands and needs. They attended courses on agrobiodiversity, identified local farmer families for seed multiplication, made and distributed the kits to other farmers. The authors opine that local initiatives based on the interconnectedness of social learning and Freire’s concepts improved food security through the practice of landrace rescue as a strategy for food security.

According to Nichols (2021) there is a proliferation of nutrition sensitive agricultural endeavors that integrate women’s empowerment and nutrition BCC components. Qualitative data collected from two projects in India implementing nutrition sensitive agriculture was analyzed to find out how women interacted with different behaviour change messages. Women were more drawn to discussions on early marriage and dietary diversity than to those on gender and health, since the latter was more complex and difficult for facilitators to communicate. The study concluded that there is an unmet need for structured spaces for rural Indian women to discuss gendered aspects of health and diet and recommended that nutrition sensitive agricultural programmes should focus on the same. The question that emerges is do agriculture and nutrition interventions, create learning spaces for critical literacy? Do they enable people to question hierarchical relations within families and societies based on caste, class and gender relations that lead to consumption of nutrient deficient diets? A nutrition literacy programme that proposes to raise critical consciousness should therefore involve the key concepts of dialogue, action and reflection, along with the creation of an open space for engaging in these activities. With this understanding of nutritional literacy, behaviour change, critical reflection and use of dialogue as a pedagogic tool, a nutrition literacy component was designed and integrated in the agricultural intervention programme.

## Methodology

**Fig. 1:**
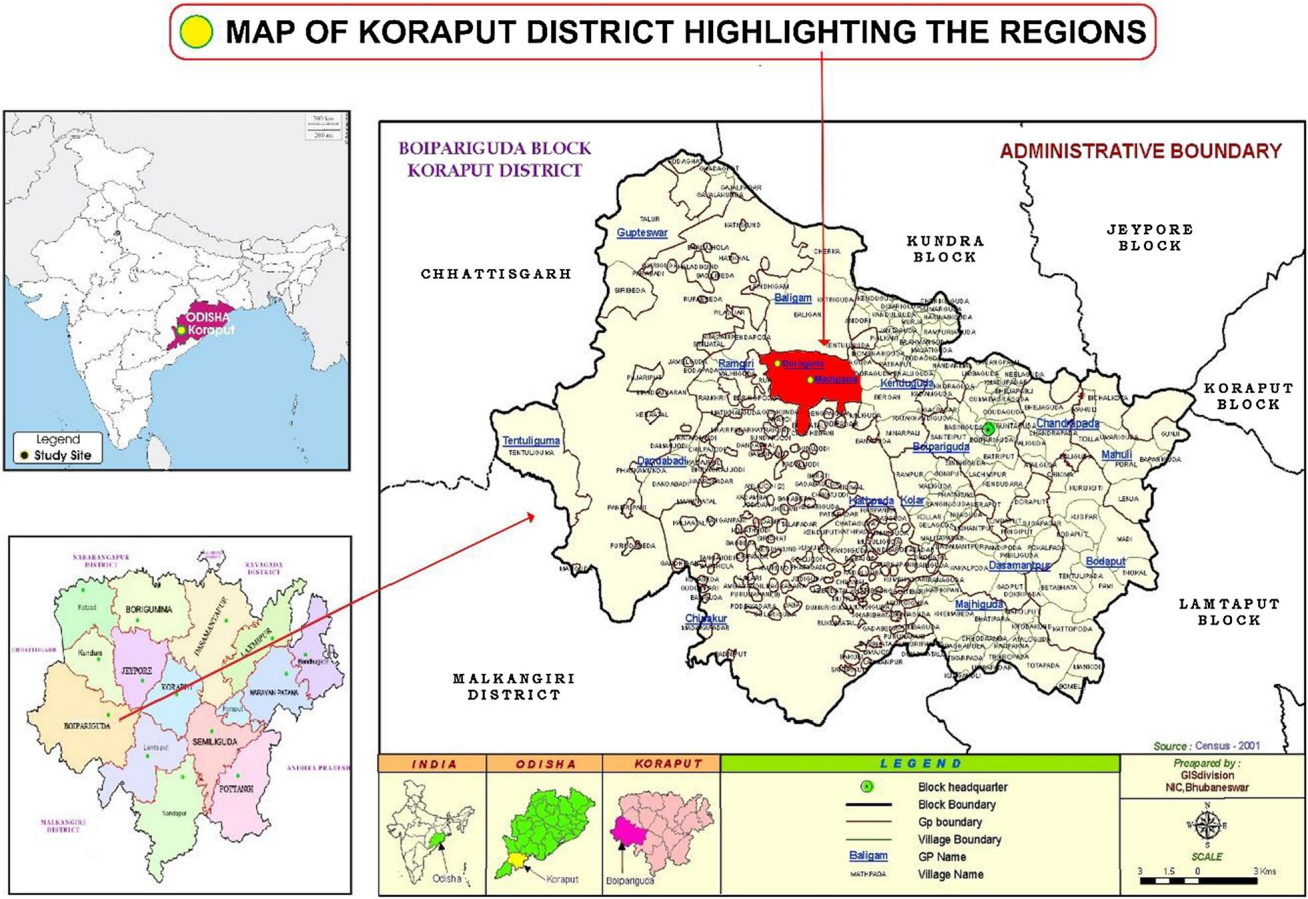
Map showing the study location

The action research for nutrition literacy is being implemented in 1575 households across 47 villages in Mathpada and Doraguda Panchayats^1^ of Boipariguda Block in Koraput district of Odisha State in Eastern India. Figure 1 shows the map of the study location.

Koraput, is a tribal population dominated district that is largely rural and rich in biodiversity; Eighty-four percent of the population live in poverty and are food insecure (DPMU, Koraput 2017); and 40.3% of children under five being stunted (NFHS 2015). The agricultural focus of the project is to enhance availability of nutrient-dense foods through increasing productivity of existing food crops and crop diversification, support nutrition gardens of fruits and vegetables at household level, backyard poultry and fish farming. A number of government schemes for agriculture and livelihood supporting in both cash and kind exist, but their effective implementation and utilization depends on the demand from the community.

In July 2018, thirty seven village meetings were held to discuss the project with 513 women and 534 men from all villages. Secondary information regarding status of agriculture, health and nutrition was presented. A few productivity trials with improved varieties and agronomic practices of finger millet, pigeon pea and orange flesh sweet potato were held. A census was taken of all households in November–December 2018 from which basic socio economic details were extracted. A baseline survey was conducted for a sample of 315 households in March–April 2019 to understand existing, agricultural systems, household food consumption pattern and nutrition awareness (summary of survey schedules provided as Additional file 1). The sample was drawn by random sampling method using SPSS statistical software (version 2).

The food items eaten by households and their frequency was collected using two methods. One was the 24 h dietary recall in which all the food items consumed by the family members, including foods eaten outside the homes were recorded for the previous day (FAO 2018). This was done for a ‘typical’ or ‘usual’ day when there was no festivity and guests. The other was the food items consumed by the household members over the past 30 days and their sources (NSSO 2014). The purpose of the surveys was to capture the diversity in food consumption at the household level and not on quantification. While a 30 day recall captures the diversity of food items consumed even if rarely, it nevertheless has the inbuilt risk of a poor memory recall since it is for a longer period of time. On the other hand memory is likely to be better under 24 h dietary recall. However one drawback of the dietary surveys was that since they were done at one point of time they are not likely to have captured the seasonal variations in food intake.

The selection of men and women to undergo training as Community Hunger Fighters (CHFs) was done at village meetings in October 2019 and 108 (59 men and 49 women) were selected. Residential training in two modules was held between November 2019 and February 2020, in batches with about 27 participants in every batch. Field observation started soon after the training, but had to be suspended between March 23rd to May 2020 due to lockdown caused by the corona virus pandemic. A second follow up was done between June and August when some relaxations were given and a third follow up between November and December 2020. Focus group discussions with 76 CHFs, consisting of 47 men and 29 women, were held between August and December 2020, spread over eight batches. Data compilation and verification from the observation, interviews and FGD reports were done between January and April 2021. While the original timeline of the project was between April 2018 to March 2021, it has been given an extension till March 2022 due to disruption in implementation.

### Process of social engagement

**Fig. 2:**
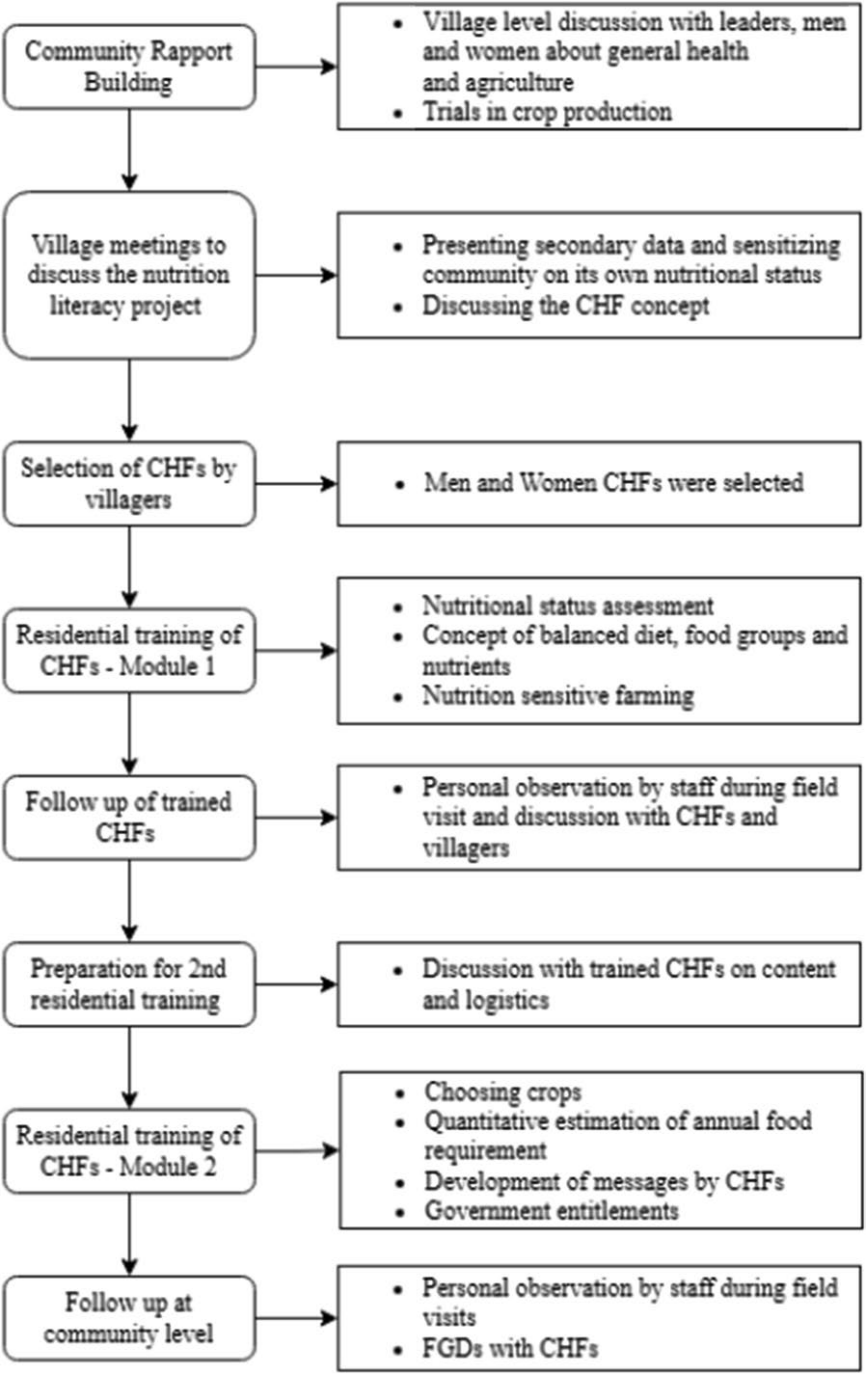
Steps in implementing nutrition literacy

Figure 2 details the steps involved in implementing the nutrition literacy programme. An external resource person with experience in implementing adult literacy programme facilitated the training. The resource person spoke in English and translation support was provided by the staff. Participants had the opportunity to stay together and share common facilities. They worked in small and large groups, interacted with and listened to the views of those from other castes, villages and gender. Unlike in real life, the residential training created space for equal participation, sharing of experiences and collective reflection about their own food and nutritional status by the CHFs. This process of working together to unpack the structural features of the experiences narrated is important since it is a way of building relationships and enhancing support, the first step towards collective decisions and action against oppression (Oakley et al. 1984). While acknowledging that one or two workshops alone would not lead to action for structural changes in the social fabric, it was nevertheless considered important to sensitize the CHFs to the issue of power hierarchy and its impact on food security. This was the first step on breaking the ‘culture of silence’ to enable the marginalized to be heard, which it was hoped would provide fodder for future discussions. The pedagogic processes used in the residential training are summarized in Table 1.

**Table 1:** Training content and methodologies

Content	Strategies for critical reflection
Understanding one’s own nutritional status	Nutritional assessment of participants and discussion on the results
Examining household diet	Small group discussion on lifestyle, daily diet and sources of food followed by presentations. Use of picture booklet as discussion starter to understand nutritive value of foods
Choice of crops and agricultural planning	Agricultural planning in small groups balancing nutritional and economic requirements in a given type of land^a^ (1 acre). The plans are discussed by all groups
Role of class, caste and gender in accessing food	Participants are randomly divided into three groups and given a situation with which to enact a role play. This is followed by a discussion and reflection on existing socio economic and cultural practices that hinder access to food by specific population groups
Estimating annual household food requirement	Participants discuss in randomly divided groups if their annual food requirement could be met with a given type of land and other strategies for augmenting food production
Introduction to Government entitlements	Face to face interaction with officials from Government departments
Identifying key messages	Village-wise group discussion

^a^Koraput is a hilly region and has 3 types of land: upland, middle land and low land

At the end of the residential training, the CHFs were encouraged to identify messages for sharing with the larger community. Beyond this point, the programme was open ended since it was anticipated that one action will flow from the other and the sequence will fold as it went along. At the individual level qualitative process indicators were developed based on participant observation—listening, seeing, questioning and analysis, to understand the process of behaviour transformation. The objective was to use observation to describe the intangible, the relationships between parts, and the underlying connections and meanings (CDRA 1998). There were eight field staff and one project coordinator. The primary source of information about individual behaviour transformation was the descriptive field notes of the staff. While some could record in English, the others recorded in Odiya which were translated by their peers. The field notes were subject to collective discussion and analysis. Verbatim statements were recorded. The questions that arose on these observations were further discussed. The observations were then grouped and classified into coded themes such as dietary diversity, food production strategies and claims for Government entitlements. Interviews with CHFs and random villagers recorded how the messages from the training had been shared. These were classified into one- on- one sharing and group sharing. In addition, focus group discussions (FGD) were held with CHFs to understand their perspectives about the transformative process. Table 2 shows the qualitative and quantitative indicators of behaviour change. The paper discusses the role of dialogue in generating critical analysis, reflection and action at the individual level.

**Table 2:** Qualitative and quantitative indicators of behaviour change

Parameters	Evidence of outcomes	Methods
CHF level	Examples of critical reflection Changes in attitudes and perceptions Sharing of information and networking with community Changes in dietary practices and food production strategies at household level	Continuous observation Interviews FGD
At the household level	Changes in food production at household level Uptake of techniques and technologies for improved food production Changes in dietary diversity and food consumption	Baseline and endline surveys
At the community level	Increased participation by marginalized groups in village level meetings Submission of claims for collective entitlements for hunger free villages	FGD with CHFs Interviews with village and community leaders, PRI members and Government functionaries

## Results

### The setting

Tribals (74%) and scheduled caste (13%) the most marginalized sections, constituted about 87% of the population. Seventy nine percent had a landholding of either less than 1 hectare or between 1 and 2 hectares and were small and marginal farmers. About 11% were landless, 8% had semi-medium (2–4 hectares) and 2% had medium (4–10 hectares) land holding. Small landholders also undertook paid labour work to augment household income. Literacy level was very low since 74% of heads of households had no formal schooling. Fifty two percent of the households had 1–4 members, 47% had 5–8 members and 1% had more. The source of drinking water was tube well for 85% of households, while another 10%, 4% and 1% sourced water from taps, open wells and surface water, respectively. Though 70% of households had toilets they were not used due to lack of water. The overall picture that emerged was one of poor subsistence farmer households with limited or no assets and lacking in basic infrastructure facilities and access to technology.

### Food intake, production and nutrition awareness

The frequency of consumption of different types of foods is presented in Table 3. Only foods eaten by atleast 25% of the population atleast once a week have been included. Rice and finger millet were the staple energy giving foods, the former being eaten everyday by all households and the latter by 85% of households. Practically all households cultivated paddy, and finger millet was grown by 62% of households. Despite 91% of households opting for improved varieties of paddy over traditional ones, production alone was not sufficient to meet household requirement and nearly 86% purchased rice from the market. Ninety percent of households had a ration card, giving an entitlement of 5 kg rice per person at Rs. 1/kg from the state run public distribution system. Since rice was a chief source of exchange for cash, households consumed only 85% of the produce and were forced to sell a portion for meeting other household expenses. With regard to finger millet traditional varieties were preferred. A majority (86%) of households also purchased finger millet from the market to meet household requirement. Oil was used daily by 57% of households for tempering. Sugar was consumed everyday by 70% of households mostly in black tea. Exposure to improved methods of food production was low. Only 14% of households had attended any type of skill development programme during the past 3 years. More than 90% of households were not aware of seed treatment for paddy and finger millet and very few practiced improved sowing methods.

**Table 3:** Frequency of consumption and source of food (n = 315)

Food item	Frequency of Consumption by households (%)					Source
	Daily	4 times a week	Twice or thrice a week	Once a week	Rarely^a^	
Cereals						
Rice	100	–	–	–	–	Own production, PDS^b^, market
Finger millet	85	6	1	1	7	Own production, market
Pulses						
Lentil	–	12	22	34	32	Market
Horsegram	–	2	8	26	64	Market, own production
Green gram	–	2	4	22	72	Market
Leafy veg						
Cabbage	–	23	28	29	20	Market
Amaranthus	–	5	18	40	37	Market
Onion stalk	–	5	12	31	52	Market
Radish leaves	–	1	5	28	66	Market
Roots tubers						
Potato	26	54	12	4	3	Market
Onion	70	11	3	2	14	Market
Radish	–	3	12	29	56	Market
Other Veg						
Tomato	74	23	3	–	–	Market
Brinjal	2	33	23	24	19	Market
Beans	–	5	22	39	34	Market
Broad bean	–	14	13	37	35	Predominantly home production
Papaya green	–	–	2	25	72	Predominantly home production
Fruits						
Banana	–	2	3	34	61	Market
Fats and oil						
Cooking oil	57	33	6	3	–	Market
Sugar	70	9	2	3	16	Market

^a^Eaten once or twice a month or never

^b^State run public distribution system providing subsidized grains

Source: Raju et al. (2020)

Pulses, the protein source were cooked as thin gruels and served as an accompaniment to rice. None could afford to eat pulses everyday, since it had to be purchased from the market. Horse gram was cultivated by 22 households and 2 cultivated green gram. Only 12 percent of households consumed lentils four times a week, though it was not grown, while it was 2% each for horse gram and green gram. About 34%, 26% and 22% households managed to consume lentils, horsegram and green gram respectively once a week. A CHF in the residential training remarked* ‘--the soil is not suitable for lentil cultivation. We buy it from the market as it is the cheapest pulse available and cooks easily. We are not familiar with cultivation techniques for green gram. Horse gram is our traditional crop but yield is poor and processing tedious. So we sell it’*. All households consuming lentils, 61% and 97% of those who consumed, horse gram and green gram respectively bought them from the market. Animal sources of protein were rarely consumed, though 80% reared poultry.

Potatoes, onion and tomatoes, purchased from the market were the most frequently consumed vegetables and formed part of the daily diet of 25%, 70% and 74% of households respectively. There was low consumption of green leafy vegetables. Twenty three percent consumed cabbage four times a week. Amaranthus, onion stalk, radish and cauliflower leaves, eaten only once a week, were also purchased from the market. Eighty percent of the households had a garden space of 15 cents (1 cent = 0.01 acre) which was operational for five months in a year, the major crops being broad bean, beans, brinjal and papaya. The frequency of consumption of home grown vegetables was also low probably due to the fact that the excess vegetables could not be stored and were either shared or sold to avoid wastage. There was an absence of fruits in the daily diet. Bananas were consumed by 34% of households once a week, mostly purchased from the market since only 6 households had banana trees in their gardens.

Dietary diversity was low with inadequate intake of protein and micronutrient rich foods. A majority (62%) of households had two meals a day. While sickness was understood, community perception about nutritional status was low, probably because the latter is invisible except at the sub clinical level. Of the 20 households that reported taking iron tablets during pregnancy 19 did not know what they were for. However in group discussions, pregnant and lactating women expressed that *‘--we know we are weak since several pregnant women who delivered in the government facility were given blood transfusion’*. Only 4 households had heard about Vitamin A. Similarly, only 18% thought it important to eat vegetables and fruits every day.

### Selection of CHFs

Household participation rate in CHF selection varied from 60 to 89% in all villages except in four in which the rate was 35%. Villagers sat in a full circle with men on one side and women on the other. Only men were involved in selecting male candidates while in selecting women both sexes were involved. Male candidates were chosen for their communication skills and whether there were other male members in the household to take care of agricultural work when they were away for training. In general, people chose individuals on whom they had faith and trust. However, gender bias as well as problems faced by women surfaced during the selection process. In Borapuda*^2^ no woman was selected since it was felt that *‘—women were not capable of communicating’*. Women were apprehensive, since they had never stepped out of their villages. Those with child care responsibilities were not chosen. Several felt staying away from home for two nights was difficult. In some villages, the lone women candidates who had been selected agreed to come only because there was another from a neighbouring village attending the programme. Literacy also played a role since several men and women in their early twenties who could read and write were selected. Some of the women CHFs were leaders of the Women Self Help (SHG) groups, while a male CHF was the President of the school committee. Several were youth leaders and one was a leader of his caste. Table 4 gives the profile of the 108 selected CHFs.

**Table 4:** Profile of CHFs (n = 108)

Gender		Caste			Literacy				
Male	Female	ST	SC	OBC	Non literate	Primary	Middle school	Secondary school	Graduate
59 (55%)	49 (45%)	84 (78%)	16 (15%)	8 (7%)	58 (54%)	19 (18%)	7 (6%)	22 (20%)	2 (2%)

## Critical reflection during training

### On adequacy of food intake and dietary diversity

In the residential training, CHFs discussed the seasonal variation in food consumption. During agricultural season only two meals were consumed since both men and women left early in the morning and came back late in the evening. It was highlighted that given the physical activity, especially for women who performed multiple roles, consuming only two meals or having a huge gap between one meal and another could lead to inadequate nutrient intake. On examining their daily diet, they realized that their food count was based on number rather than variety (e.g.) if someone consumed rice, finger millet gruel, potatoes and maize, it was seen as adequate since there were four food items. When the role of different food groups in maintaining health was discussed, one CHF remarked *‘—we never thought about having both dhal and vegetables in the everyday diet. So long as one was there as accompaniment to rice we were satisfied.’*

### Perception of cultural loss in relation to specific foods

In another exercise the annual food requirement of a household comprising of three adults and two children was estimated based on the recommended daily allowance (RDA-provided as Additional file 2) for Indians (NIN 2011). Ten quintals of rice were needed annually. Working in three groups, CHFs calculated how far the requirement could be met with a kitchen garden and one acre of upland, middle land and low land respectively. Participants estimated that only 3 and 7 quintals of rice could be produced from upland and middle land respectively. The shortfall could be partially met through the subsidized rice provided through the PDS. Cash crops such as cashew and little millet could be grown and sold, with which additional rice could be purchased from the market. Only lowland yielded about 18 quintals of rice, eight more than the annual requirement. It was not surprising therefore that the choice of paddy variety was based on yield and marketability over taste or tradition. Two factors influenced rice cultivation—the replacement of traditional varieties by hybrid ones since the latter gave higher yield and were procured by the government. While traditional varieties are climate smart, resistant to pest attack and are valued for their organoleptic properties (Mahapatra 2020) they lose out when pitted against high yielding varieties. As one woman CHF lamented it was also a cultural loss—*‘There used to be a specific song for each traditional variety of rice and now that the rice varieties are disappearing the songs are also being forgotten’*. They were further concerned that tribal children who studied in hostels refused to eat finger millet gruel, their traditional food when they came home.

### Gender and social norm and nutritional inequity

The nutritional assessment was useful in helping CHFs to understand that physical and biochemical features can be useful technological tools in understanding about one’s health. Based on heights and weights, the Body Mass Index (BMI) was calculated and one third of the CHFs, most of whom were women were found to be severely undernourished. While all were anaemic, 62% women and 10% men had severe anaemia. All were given dietary advice and adviced to collect iron and folic acid tablets from the local health workers. This experience affected men and women differently. *Pani,* a woman CHF was moved to tears. According to her *—‘no one has been concerned about my health till now. I was the one always taking care of others in the family’*. For the male CHFs, it was a revelation that women were so undernourished.

The gender difference in nutritional status was attributed to less access to nutritious food by women due to socio cultural factors. Even during the residential training women belonging to the *Bhumia* and *Rana* castes would not eat the food prepared in the common kitchen due to caste taboos. They only ate raw, uncooked and dry food. They were also forbidden from eating meat while the men could do so. On attaining puberty, girls were given only cooked rice and jaggery for about ten days. Women said that it would be impossible for them to defy caste restrictions as they would be fined if they did. Existing social norms could be changed only with the active involvement of men. Women’s multiple work roles and long hours of work with less access to nutritious foods emerged from the role play. Men agreed that sharing of household work is essential for relieving women from laborious and drudgery ridden work. They identified tasks that they could do such as fetching water and childcare to which one woman CHF replied *‘—Let us see if you live up to your promises’*. Another area of concern was that school children who stayed in State run tribal hostels, were averse to eating the traditional ragi gruel when they came home.

### Nutrition sensitive agricultural planning for dietary diversity

In the agricultural planning exercise, participants showed that upland could support four types of food groups. Besides cereals, pulses could be grown as mixed crops or in the residual moisture after harvesting of rice. A variety of green leafy and other vegetables could be grown. In the bund mango and banana trees could be planted. The area was divided into plots of varying sizes and one cash crop such as cashew along with food crops was planned to balance both nutrition and economic return. With regard to middle land, short duration rice varieties could be grown. After draining out excess water from the land, green leafy vegetables, radish, sweet potato, coriander, papaya and ladies finger could be raised. In the bund ridge gourd and cow pea that required less moisture could be planted. Overall three or four food groups could be cultivated.

In low land though cultivation was not possible in rainy season due to water logging, five varieties of food crops could be raised. Besides rice, pisciculture could be taken up. With a farm pond, root, green leafy and other vegetables could be produced along with fruits such as banana, papaya and water melon. In the kitchen garden, CHFs identified about 25 vegetable and fruit crops that could be raised along with a commercial crop such as tamarind. Poultry could also be reared. In summer, when water scarcity was acute, participants said that household waste water could be used to water the plants. Usually vegetables produced from the kitchen garden were consumed while those grown in the field were sold. Henceforth they proposed to use a portion of the latter also for home consumption.

### Identifying limitations, bottlenecks and recognizing the state role

While estimating annual pulse production to meet household requirement, CHFs calculated, a shortfall of about 30 kgs for upland and middle land, while it could not at all be grown in lowland. Though several strategies to improve production were identified, the CHFs felt that part of the requirement could be met only through purchase from the market. Additional money had to be earned through labour work. Participants felt that even then, pulses could be consumed only for about three or four days a week. As far as finger millet was concerned, upland could yield 100 kgs which was much higher than the annual requirement of 72 kgs. For middle and low land the shortfall was between 30 and 40 kgs that had to be purchased from the market.

Lack of water supply for irrigation was cited as a major problem in food production. Several households had applied for constructing farm pond and dug well under the Rural Employment Guarantee Scheme. Some who had obtained the contract and completed the work were yet to receive payment. Representations to the concerned authorities yielded no result. Seeing this several others hesitated to take up the work. As one CHF put it ‘*we are poor, non-literate and do not know how to engage with the Government*’. The session on the various Government schemes was the first instance of *‘an equal and meaningful engagement between us and the Government’* as articulated by one CHF. It was discussed that one way to address this was to collectively undertake the work through village committees which would engage with the government. The poorest should have adequate representation in such committee.

### Deriving key messages from the training

At the end of the first module, participants were requested to identify a few key messages that they would like to share with their community. Upon deliberation, they developed three messages.

- Eat three to four types of food groups everyday for maintaining good health.
- Carry lunch to the field to avoid huge gaps between meals.
- Let us share household work and reduce the burden on women.

While the first dwelt on bringing in dietary diversity in the daily diet, the second dealt with lifestyle modification and the third focused on gender equity.

### Identifying the way forward

The CHFs identified further training needs for improving food production that included seed treatment and sowing techniques for paddy cultivation, package of practices for home garden, pulse cultivation, pisciculture and integrated pest management using organic methods. They wanted the training programmes to be conducted at the village level with a larger group of farmers. However due to total lockdown from March 2020 project activities were suspended. With gradual relaxation of the lockdown from September 2020, exposure visits were organized for a limited number of participants to see activities of biofortified rice crop cultivation, pisci culture, nutrition garden and crop management using organic methods. Agricultural trainings were conducted in the open at the village level with due adherence to COVID protocols. Training activities had to be suspended again between December 2020 and January 2021 due to crop harvest and were resumed in February 2021.

## Co-construction of knowledge during training

The pedagogic approach facilitated in building on the existing knowledge of the CHFs. In nutrition sensitive agricultural planning, participants working in groups, drew the map of an imaginary field showing the cultivation plan and strategies for augmenting production for different types of crops. In rice cultivation when traditional broadcasting method was discussed, the staff suggested the use of line sowing method for improving production. In other instances they suggested growing crops in the bunds, alternate foods that could be grown and improvement in the layout. As they went around discussing, the participants challenged, questioned, sought clarifications and contributed to improving each other’s plans. They considered the shared learning to be *—‘interesting, useful and enjoyable’*.

## Action by CHFs

The CHFs were followed up with by staff members to observe the changes following the training.

### Networking and sharing

Hundred CHFs had conveyed the messages to families, friends and neighbours through formal and informal means. Seventy five percent had shared all the three messages. Mootha Kadki*^3^ of Kendamal* village had shared it with 28 persons from the neighbouring village in the weekly market. One CHF, a priest disseminated the information in the temple. The CHFs from *Mesapud* had shared the messages during harvest while those from *Tantlipud* had done so in the tea stall and threshing yard. *Chapa Santa* of *Gopada* had visited all the houses in her village as well as in neighbouring villages to personally share the messages and so did *Dhan Kher* from *Bargud* village. While sharing informally, a few CHFs had even bent the existing social norms. In *Mesagud* and *Delpar* villages two female CHFs belonging to the upper caste group had visited the SC street and ST street, respectively. In *Mesapud* male CHFs made a presentation in the women’s SHG group, which was a break from regular practice.

**Fig. 3:**
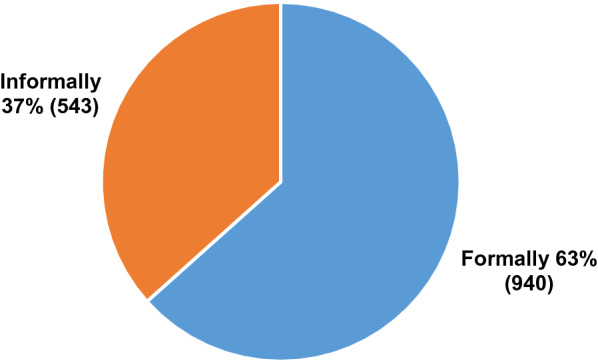
Percentage of people contacted through formal and informal meetings

In formal sharing the CHFs had adopted two strategies. One was to use the existing grassroot structures and the other was to convene special village meetings. Women CHFs found the SHG meetings to be ideal forums for dissemination. In *Murgagud* the villagers meet regularly on the 15th of every month to discuss village issues and the CHFs found this to be a convenient forum. A male CHF who is the leader of the *Gadaba* tribe said that he had made a presentation in his community group meetings outside of his own village. Between mid November 2019 to January 2020, 52 CHFs had organized 66 meetings, of which 40 were at the village level, 22 were women’s group meetings and two at the Panchayat level, together reaching approximately 1483 persons. Formal meetings were organized collaboratively. In Nishnapuka* all the four CHFs had jointly organized the meeting. As one CHF remarked *‘—initially we were scared but now after several meetings we are more confident and better at addressing people’.* Women CHFs took the help of male CHFs or other male leaders in the community to convene the meetings. About 63% of villagers had been reached through formal meetings while 37% through informal meetings (Fig. 3).

Responses to the messages were mixed. There was greater receptivity to the messages at the household and SHG levels. The CHFs of Nishnapuka* said that 60% of the meeting participants said that achieving dietary diversity was doable while 40% were skeptical. The young CHFs who had shared the messages in the youth club said that members wondered if it would ever happen in their villages. The male CHF who was the leader of his tribe said that *‘––– in the meetings people asked me ‘from where are you getting all this information?’*. In Murgagud* the CHFs said that people from their own caste (SC) had been skeptical whereas those from the ST group were more receptive. However in Meligud* where there was no water scarcity participants felt they should take up round the year cultivation. The bottlenecks such as lack of irrigation facility, an apathetic government and fear of breaking social norms, also raised by the CHFs in the training were identified as impediments to achieving nutrition security.

### Behaviour transformation

There was a wide range in the diversity of actions taken by the CHFs to achieve food security. The starting point for each CHF was different commensurate with their age and position in their households. All CHFs reported to consuming three meals a day. Twenty CHFs had started taking food to the field. The male CHF of Bindraged* had requested his mother to carry food when she was spending the whole day in the field. She had replied *“—I cannot do so since I am already burdened with carrying agricultural equipment*’. He had started packing her lunch and sending it regularly through his father. Thirty eight CHFs had revived their kitchen gardens. Nina* from San Anar* had planted green leafy vegetables while Mita* from Tendamal* had sown several varieties of vegetables and fruits in her home garden. Mita* has also expressed her desire to set up mother nursery of Orange Flush Sweet Potato. Krish Matpud* of Nugud* has designed and adopted a nutrition garden throughout the year for daily supply of vegetables and fruits. The actions of the CHFs had impacted other households in some villages. Ten households from Tentapad* and Harguda* upon seeing the CHFs had set up a kitchen garden. Dasth Kisni* and Parti Pang* of Nishpak* village said that a discussion had been generated in their village about the need for diversity in everyday diet, but many were initially reluctant to set up a kitchen garden. However, after seeing the two CHFs they had started taking an interest. There was no appreciable increase in the consumption of animal food since they were maintained more for income through sale.

The emergence of leadership from CHFs who had hitherto not been in such a position was another dimension witnessed. Several CHFs had started engaging with the community on other issues of importance. During lockdown, all CHFs set an example by following the State guidelines of masking, hand washing and maintaining social distancing. Bhagti Pan* of Tipgud* spread awareness on COVID guidelines, and Chepa*, a woman CHF from Gopad* voluntarily participated in the malaria awareness programme and reached out to three villages. During COVID Alba* of Murjagud* urged all households to utilize the cash support given by the government for food security. Pat Khar* from Dalpu* distributed the fish harvested from the community pond as well as sale proceeds to all needy households during lockdown. However there was no respite for women from household chores which had increased twofold during lockdown.

### Further reflections

In the FGDs, the staff and the CHFs shared and discussed their views and experiences and reflected on the immediate and long term actions to be taken.

1. Achieving household food security throughout the year is a long term goal. However despite being poor and marginalized, a number of immediate changes can be brought in at the household level to improve the existing situation.
2. Long term action will call for creation of adequate resource and infrastructure in the villages for which there needs to be engagement with the government. It will be useful to develop a village action plan to this effect.
3. In engaging with the community, the use of tools such as the picture booklets distributed in the training are very useful since they help concretize the messages and give authenticity.
4. Collective action is important and this was strongly realized during the COVID pandemic. There was a heavy setback to household food security during lockdown in April and May of 2020 due to economic distress created by lack of labour work for landless and migrant households. Since this was the lean agricultural period landed households were less affected. However, about 10% of households that had access to water and undertook cultivation of rice during this period faced problems of lack of labour and access to fertilizers due to closure of market leading to less production. Despite State initiative through free distribution of food grain and provision of Rs. 1000 in cash, deposited in women’s bank accounts in two instalments and community kitchens set up at *Panchayat* level about 207 households had foraged food from the forest and 155 had faced hunger, between April to September 2020. There was no collective community response to the situation.
5. Several CHFs with no prior experience of formal leadership expressed that the intervention had given them an opportunity to discover their own potential for leadership, whether it be in organizing meetings or in tackling the COVID situation.
6. Social norms, especially gender inequity are harder to address and will take a much longer time. In general, community behaviour transition is possible only when there is sustained effort at sensitization.

## Discussion

**Fig. 4:**
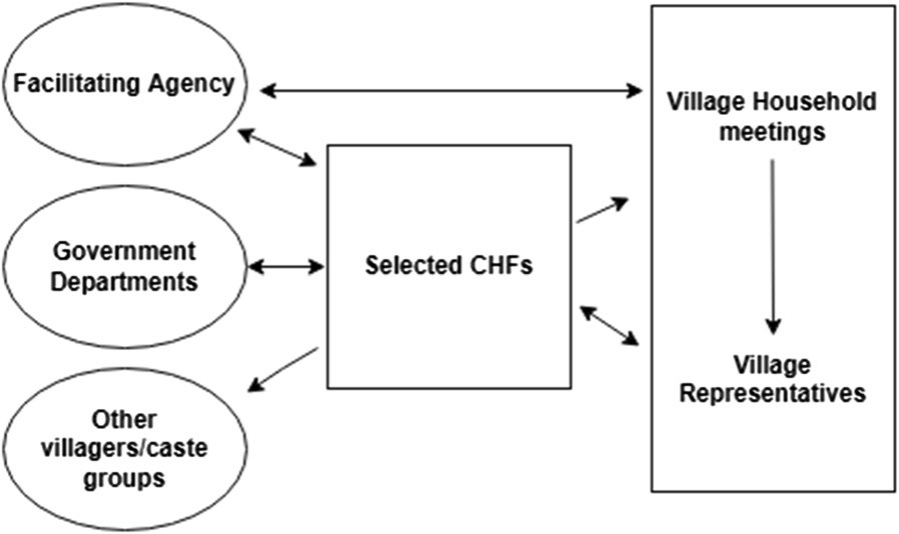
Dynamics of dialogue

The use of continuous dialogue as an engagement process brought dynamic exchanges between various stakeholders and generated a discussion on household food and nutrition security in the study villages (Fig. 4). The reflection exercises in the classroom enabled CHFs to identify the social, cultural and political barriers to household food security. Of particular interest was the shift in perceptions over gender inequity. The sensitization about women’s poor nutritional status, their multiple work roles with lack of support and the social control imposed on their food intake raised the question and discussion on ‘should it be so?’. Male CHFs accepted that patriarchal social norms needed to be addressed. This shaped the message on ‘support to women’ to be shared with the community. Mezirow (1997) theorizes that a change in the frame of reference occurs when there are critical reflections and dialogue on long held assumptions disproved by evidence. This in turn leads to autonomous responsible thinking. Subsequent actions by male and female CHFs in taking a leadership role in jointly convening village meetings, or acting as resource persons in women’s SHG meetings are evidences of such transformation. This justifies the project’s stand that sensitizing men about women’s powerlessness is essential to challenging patriarchal norms. On observing that women recalled messages better over which they could take action and resonated less with those over which they felt powerless. Nichols (2021) makes a strong case for including men in participatory endeavours. For women who had taken their nurturing role for granted the feeling of being cared for was a moving experience. Using quantitative and qualitative data, Niehof (2019) observes that ‘—because of women’s hegemonic role in family food care and their often being relationally defined as mothers, their own nutritional needs tend to be considered instrumentally rather than as a matter in its own right’.

Male perception about women’s lack of communication skills manifest during the selection of CHFs was a stereotyping of their agency. Hentschel et al. (2019) while studying about the multiple dimensions of gender stereotypes among men and women belonging to various racial groups observe that, male raters described women to be lower in most aspects of agency when compared to men; so did female raters who considered men as more competent leaders. The women SHG movement in India, more than three decades old, was originally started as a savings and credit group and later expanded to include addressing gender and caste based discrimination (Kumar et al. 2021). However rather than transforming power structures they have been reduced to producing responsible financial subjects or dutiful mothers (Batliwala 2007; Narayanan and Rao 2019). While women SHGs have emerged as promising platforms for delivery of nutrition sensitive agricultural programmes, there is unmet need among Indian rural women for structured spaces to discuss gendered aspects of health and diet (Nichols 2021). Following the residential training all the women CHFs had started discussing about household and village food security in the SHG meetings.

The other social norm challenged was caste segregation. Upper caste CHFs visited lower caste households to share training experiences. This was probably due to the effort taken to create an ‘open space’ without barriers for learning. Open space offers an integration of cognitive, affective and experiential forms of learning that promotes self-discovery and transformative growth (Lee et al. 2021). In the process of learning it is important to help participants form an honest picture of themselves and to value their own strengths that they would not like to loose (CDRA 1998). The CHFs were nostalgic about the loss of traditional land races and the change in food preferences of the younger generation whose food habits were being moulded by State hostels. The need to go beyond the boundaries of one’s own village and engage with the State was recognized. However past engagement with the State had not been successful. The need for a collective forum for engaging amongst themselves and with the Government was realized during lockdown, when individual households struggled to cope with loss of food security. While reality is often complex and beyond immediate grasp, connections become clearer in due course, to enable addressing them in a holistic manner (CDRA 1998).

Since there was no prescriptive approach for behavioural change, individual CHFs were encouraged to charter their own course towards food security, resulting in diverse actions. Peoples’ participation is not passively taking part in activities designed by others; It is the taking of initiatives to decide what is to be done and how to do it (Oakley et al. 1984). CHFs not only spread messages about dietary diversity but also attempted to practice them in their own ways. Transfer of learning occurs only when learners use the new concepts or skills in their regular working or life context (Burke and Hutchins 2007). For such a transfer to occur the process of learning must be ‘scaffolded in the social context’, which does not happen in conventional educational programmes (Roumell 2019), due to which there is little change after initial learning. By transforming themselves the CHFs set an example to their community. A few households after seeing them set up a kitchen garden, proceeded to do so themselves.

This paper offers a methodological contribution to how Freire’s theories on adult learning may be integrated in a nutrition sensitive agricultural intervention programme for achieving dietary diversity. One limitation is that the project is a short term one for a three year period while long term interventions are needed to study sustainability and insights for replication at scale.

### The way forward

Relationship building and personal interaction are at the core of using critical literacy and dialogue as a pedagogic tool in adult interventions. These were challenged during lockdown, leading to looking at alternate strategies of engagement. To some extent these were addressed by working in smaller groups and having short one on one discussions through cell phones. However use of other communication strategies such as community radio and video screenings for technical trainings need to be explored when faced with similar situations in future. There is already a demand for refresher training from the CHFs. An interface between government departments and CHFs will be organized to evolve modalities for continued engagement of the local community to operationalize the concept of hunger free villages.

## Conclusions

The use of dialogue as a pedagogic tool in critical nutrition literacy, that is embedded in the social, cultural and political environment of the participants has the potential to enable them to derive their own meaning and messages suitable to their context. It could also lead to behavioural transformation. An open classroom with no prescriptive approach and where knowledge about nutrition sensitive agricultural planning was co-constructed led to shared learning which was extended to the larger community. Critical reflections on daily food intake and production offer insights on general preferences, issues in diversifying food production, cultural loss in relation to specific foods and gendered nature of nutritional inequalities. Future endeavours should consider engaging with a mixed group of participants consisting of both men and women, since discussion between the two sexes is effective in sensitizing men to gendered disparities in agriculture and nutrition. Resource persons with experience in critical literacy for adults would be required to guide the programme. Long term interventions are needed to study the sustainability of such programmes.

## Supplementary Information

**Additional file 1: **Summary of the survey questionnaires.

**Additional file 2: **Recommended Dietary Allowances (For adult male).

## Data Availability

The datasets used and/or analysed during the current study are available from the corresponding author on reasonable request.

## Footnotes

1 Unit of local self-governance consisting of elected representatives.

2 *Names changed for protecting identity.

3 * Names changed for protecting identity.
