# Supplementary material for: Dialogue as a tool of nutrition literacy in an agricultural intervention programme in Odisha, India
Source: CABI Agric Biosci. 2022 May 10;3(1):28. doi: 10.1186/s43170-022-00090-x (PMC9088138; doi:10.1186/s43170-022-00090-x)
Supplement: Supplementary file 1 — Additional file 1: Summary of the survey questionnaires. [file 43170_2022_90_MOESM1_ESM.docx]

**Additional file 1. Summary of the survey questionnaires**

**Schedule 1. Household details.**

The schedule collected details on the following :

1) Household id 2) name of the village and Gram Panchayat to which it belonged 3)name of respondent and id 4) Caste 5) Religion 6) total no of members and their details regarding age, sex, schooling, marital status, occupation 7) land holding and type 8) whether land is leased out/fallow 9) ownership of livestock 10) backyard / farm pond if any 11) housing details (if own or rented, whether kutcha or pucca (based on material used for construction) 12) source of drinking water 13) availability of toilet 14) principal source and annual income of households 15) if having ration card and if yes type of card 16) if member of any grass roots institution such as SHG, village development council etc 17) whether having bank account.

**Schedule 2. Agriculture, Home garden and Fishery details**

1. Household farm details in *Kharif* (June to November 2018) and *Rabi* (December 2017 to May 2018) seasons. (total operational land includes own as well as leased)

| Land type | Crop name | Area (in acres) | | | Total output (qtl. kgs) | Self consumption  (qtl. Kgs) | Amt sold  (qtl.kgs) | If sold price  (rs) | Amt  Stored  (seed)  kgs | Remarks |
| --- | --- | --- | --- | --- | --- | --- | --- | --- | --- | --- |
|  |  | Irrigated | Rainfed | Total |  |  |  |  |  |  |
| Upland |  |  |  |  |  |  |  |  |  |  |
| Middle |  |  |  |  |  |  |  |  |  |  |
| Lowland |  |  |  |  |  |  |  |  |  |  |

2. Vegetables/ fruits grown in home gardens (January to December 2018) – total area, no of months for which home garden was operational, vegetables/fruits grown, total output, quantity used for home consumption, quantity shared with community members, amount sold and price.

3. ***Agricultural practices***

a) awareness and practices about land preparation – details thereof

b) seed practices for rice, finger millet, maize, pigeon pea, green gram, black gram – variety grown, sourcing, seed treatment and storage practices

c) sowing methods for the above crops

d) awareness and practices about nutrient management

e) integrated pest management practices

f) value addition if any

g) marketing details for paddy, finger millet, maize, little millet, pulses

4. Household Fishery details – presence of a fish pond, area, operational period, type of fish reared, total output, amount consumed, shared, sold and price if sold. Fish farming management practices, feed used for aquaculture

5. Poultry and Meat – number of hens (adult female) production of eggs, meat, quantity consumed, shared, sold and price if sold.

6. Production, consumption and sale if any of mushrooms.

7. Whether has received skills training during the last 3 years on any of the following : seed treatment, improved agronomic practices, Integrated Pest Management, Integrated nutrient management, seed selection, value addition, vermi composting, mushroom cultivation.

**Schedule 3. Household Food Consumption Pattern**

Reference period – previous one month

| **Food group*** | **No of days consumed in a month** | **Source**  **(use codes)**** | **If bought, market price (rs)** | **Remarks** |
| --- | --- | --- | --- | --- |
| Cereals and millets |  |  |  |  |
| Pulses and legumes |  |  |  |  |
| Leafy vegetables |  |  |  |  |
| Roots and tubers |  |  |  |  |
| Other vegetables |  |  |  |  |
| Nuts and oilseeds |  |  |  |  |
| Fruits |  |  |  |  |
| Fish and sea food |  |  |  |  |
| Meat and poultry |  |  |  |  |
| Milk and dairy products |  |  |  |  |
| Fats and oils |  |  |  |  |
| Sugar |  |  |  |  |
| Wild foods |  |  |  |  |

* under each heading an exhaustive list of food items was provided. Those not covered in the list were manually added at the time of survey.

** codes were provided for different sources and their combinations such as home production, market, public distribution system, forest, borrowing from neighbours, friends, family

**Schedule 4. Household diet survey**

Note: make sure households did not have guests, feasted or fasted the previous day. Ask the respondent what the family members ate from the time they woke up till they went to sleep the previous day. Include food consumed outside the home

| **Time** | **Name of the dish** | **Ingredients used to prepare the dish** | **Remarks (mention if eaten outside the home)** |
| --- | --- | --- | --- |
| Early morning |  |  |  |
| Breakfast |  |  |  |
| Lunch |  |  |  |
| Evening |  |  |  |
| dinner |  |  |  |
| Bed time |  |  |  |

**Schedule 5. Nutrition awareness**

There were questions on what should our daily diet include, what is the importance of fruits and vegetables, whether heard about anaemia, it’s symptoms, sources of iron rich foods, whether heard about Vitamin A deficiency, sources of Vitamin A /carotene rich foods, symptoms of Vitamin A deficiency, hand washing practices, safety treatment for drinking water if any, supplement given by government for pregnant women, breastfeeding and complementary feeding practices, importance of first 1000 days of life, whether received any health messages and their source.
