# Supplementary material for: Dialogue as a tool of nutrition literacy in an agricultural intervention programme in Odisha, India
Source: CABI Agric Biosci. 2022 May 10;3(1):28. doi: 10.1186/s43170-022-00090-x (PMC9088138; doi:10.1186/s43170-022-00090-x)
Supplement: Supplementary file 2 — Additional file 2: Recommended Dietary Allowances (For adult male). [file 43170_2022_90_MOESM2_ESM.docx]

**Additional File 2. Recommended Dietary Allowances (For adult male)**

| FOOD GROUPS | RDA(g/ per day) |
| --- | --- |
|  |  |
| Cereals & Millets | **375** |
| Pulses & Legumes | **75** |
| Green Leafy Vegetables | **100** |
| Roots & Tubers | **200** |
| Other Vegetables | **200** |
| Fruits | **100** |
| Milk & Milk Products | **300** |
| Fats & Edible Oils | **25** |
| Sugar & Jaggery | **20** |

Source: NIN, ICMR, 2011
